# Investigation of Inflammatory Markers in Patients with Polycystic Ovary Syndrome Receiving and Not Receiving Metformin Treatment

**DOI:** 10.3390/medicina61061012

**Published:** 2025-05-28

**Authors:** Aykut Bulu, Erhan Onalan, Burkay Yakar, Gülay Bulu, Senanur Onalan Yıldırım, Kader Uğur, Emir Dönder

**Affiliations:** 1 Department of Internal Medicine, Fethi Sekin City Hospital, Elazig 23119, Turkey; dr.aykutbulu@hotmail.com; 2 Department of Internal Medicine, Firat University, Elazig 23119, Turkey; e.donder33@hotmail.com; 3 Department of Family Medicine, Firat University, Elazig 23119, Turkey; byakar@firat.edu.tr; 4 Department of Gynecology, Fethi Sekin City Hospital, Elazig 23119, Turkey; gulay.figen@hotmail.com; 5 Department of Gynecology, Firat University, Elazig 23119, Turkey; senanuronalan8@gmail.com; 6 Department of Endocrinology, Firat University, Elazig 23119, Turkey; kaderaksoy06@hotmail.com

**Keywords:** polycystic ovary syndrome, inflammation, C-reactive protein, NLR, PLR

## Abstract

*Background and Objectives*: Polycystic ovary syndrome (PCOS) is one of the most common endocrine disorders in women of reproductive age. The etiology of PCOS is complex and is associated with low-grade chronic inflammation. The current study aimed to investigate inflammation markers in PCOS patients with and without metformin treatment. *Materials and Methods*: This cross-sectional study included 30 age-matched PCOS patients not receiving metformin treatment, 50 PCOS patients receiving metformin treatment, and 30 healthy controls. The groups were compared according to inflammatory (hs-CRP, NLR, and PLR) and metabolic parameters (lipids, fasting blood-sugar insulin, HOMA-IR). *Results*: Insulin (*p* < 0.001) and HOMA-IR (*p* < 0.001) score median values of PCOS patients were found to be significantly higher than the control group. CRP levels of PCOS patients receiving metformin treatment were found to be higher than both control and PCOS patients not receiving metformin treatment (*p* < 0.001). There was a significant difference between the groups in terms of PLR mean value (*p* = 0.031). The mean PLR value of PCOS patients, both those receiving metformin treatment and those not receiving treatment, was found to be significantly higher than the control group. In PCOS patients not receiving metformin treatment, there was a negative significant correlation between NLR and HDL level (r: −0.384; *p*: 0.036), NLR and insulin (r: 0.422; *p*: 0.020), and HOMA-IR score (r: 0.439; *p*: 0.015). There was a positive significant correlation between them. *Conclusions*: In the current study, PLR was significantly increased in all PCOS patients compared to controls. CRP levels in PCOS patients receiving metformin treatment were significantly higher than both control and untreated PCOS patients. PLR is positively associated with insulin and HOMA-IR scores in PCOS patients.

## 1. Introduction

Polycystic ovary syndrome (PCOS) is one of the most common endocrine diseases in women worldwide. PCOS affects 10% to 13% of women [1]. Clinically, hyperandrogenism is characterized by an increased risk of anovulation, infertility, psychosocial dysfunction, and metabolic disease. The etiology of PCOS has not been clearly elucidated. A familial genetic syndrome resulting from a combination of environmental and genetic factors is the most accepted theory [2].

Weight gain, abdominal and subcutaneous fat, hirsutism, male pattern alopecia, clitoromegaly, deep voice, seborrhea, and acne, which are symptoms of hyperandrogenism, are the most common symptoms of PCOS. In addition to these morphological features, changes also occur in the metabolic profile. Insulin resistance is the most common metabolic symptom of PCOS. This condition results in hyperinsulinemia and can lead to diabetes. High insulin levels are responsible for the accumulation of fat around the abdomen. Most women with PCOS have a body mass index (BMI) of 30 or higher. Apart from this, hypertension, cardiovascular problems, and dyslipidemia are diseases accompanying PCOS [3].

Previous literature data have reported that in addition to hyperandrogenism and metabolic disorders, there is an inflammatory process in the body in individuals with PCOS. Mean platelet volume (MPV) and neutrophil/lymphocyte ratio are higher in PCOS patients than in healthy women. Leukocytosis, neutrophilia, and platelet aggregation are frequently detected in the blood profile of PCOS patients [4]. Serum IL-6 levels have also been shown to be elevated in women with PCOS compared to controls. Levels of CRP (C-reactive protein), a measure of inflammation, are elevated in PCOS patients. The relationship between high CRP and diabetes, atherosclerosis, cardiovascular diseases, and other inflammatory diseases is known. The release of inflammatory markers is associated with long-term metabolic complications and increased cardiovascular risk [5,6].

Physical activity and diet are the first therapeutic approach for PCOS patients. In addition to lifestyle changes, metformin and combined oral contraceptive (COC) therapy are the most common medical treatments. PCOS treatment aims to improve weight, insulin resistance, hyperandrogenism and the inflammatory state frequently seen in PCOS [7]. Both metformin and COCs have an antihyperandrogenic effect and thus can suppress the inflammatory process. Despite all these treatments and pathophysiological processes, no definitive drug or definitive treatment has been approved for PCOS [8]. The effect of current treatment approaches on the inflammatory process is not clearly known.

This study focused on the relationship of metformin treatment with the inflammatory process in PCOS patients. This study aimed to compare some inflammation markers in PCOS patients who received and did not receive metformin treatment and to examine the relationship between treatment and inflammation marker levels.

## 2. Materials and Methods

This cross-sectional study was conducted with individuals receiving outpatient treatment in the endocrinology clinic of a tertiary healthcare institution between January and March 2024. The study population consisted of patients diagnosed with PCOS who applied to the endocrinology clinic. PCOS patients who did not receive medical treatment, PCOS patients who received metformin treatment and healthy controls were included in the study. Female patients aged 18 and over were included in the study on a voluntary basis. Sample calculation was carried out with open access Web-Based Sample Size and Power Analysis Software (https://biostatapps.inonu.edu.tr/). While the amount of Type I error (alpha) is 0.05, the power of the test (1-beta) is 0.8, and the effect size is 0.41, the minimum sample size required to find a significant difference using the independent samples *t*-test should be 63 in total, 21 for each group. A total of 30 healthy controls who met the study criteria, 30 PCOS patients not receiving metformin treatment, and 50 PCOS patients receiving metformin treatment were included in the study. The study sample was randomly selected from patients with PCOS and healthy controls who applied to the endocrinology clinic.

Inclusion criteria for the healthy control group were: being over 18 years of age, volunteering to participate in the study, and not having any known metabolic or chronic disease. Those with acute infection, known metabolic or chronic disease, receiving hormonal treatment, obesity, and menstrual cycle irregularities were excluded from the study.

Patients who met the following criteria were excluded from the study: (1) other diseases related to or affecting PCOS (e.g., hyperprolactinemia, congenital adrenal hyperplasia, thyroid disorders, Cushing’s disease, various cancers); (2) hepatic or renal dysfunction; (3) women taking antiandrogens, antidiabetics, lipid-lowering drugs, glucocorticoids, or other hormonal drugs; (4) inability to communicate in the local language; (5) poor attitude and refusal to cooperate.

PCOS was diagnosed by meeting all the Rotterdam PCOS diagnostic criteria (oligo- or anovulation, clinical or biochemical hyperandrogenism, and polycystic ovaries on ultrasound) [9]. PCOS was diagnosed with the presence of at least two of these three criteria. Women used as controls were recruited based on the same inclusion criteria of age and BMI (≥27 kg/m^2^) as women with PCOS. Controls were also required to have a regular menstrual cycle of 23 to 32 days. The body mass index (BMI) of the participants in the study was calculated using the formula weight (kg)/height square meters (m^2^). Following an overnight fast on the second or third day of the menstrual cycle, venous blood samples were taken from all participants for biochemical and hematological analyses. The samples were immediately sent to the hospital laboratory, and the necessary analyses were performed. From the samples taken, estradiol (E2) (pg/mL), FSH (mIU/mL), LH (mIU/mL), dehydroepiandrosterone sulfate (DHEA-SO_4_) (μg/dL), TSH, low-density lipoprotein (LDL) (mg/dL), high-density lipoprotein (HDL) (mg/dL), triglyceride (TG) (mg/dL), total cholesterol (TC) (mg/dL)/dL), fasting blood sugar (FBS) (mg/dL), high-sensitivity C-reactive protein (hs-CRP) (ng/mL), insulin (IU/mL), BUN, creatinine, AST and ALT were measured. Insulin resistance was calculated with the formula glucose (mg/dL) × insulin (μIU/mL) × 0.056/22.5. Hematological parameters, such as “white blood cell, hemoglobin, neutrophil, lymphocyte, platelet”, were obtained from hemogram analysis. Neutrophil lymphocyte ratio (NLR) was defined as “Neutrophil count/Lymphocyte count”, and platelet lymphocyte ratio (PLR) was defined as “platelet count/Lymphocyte count”.

SPSS 22 (Statistical Package for Social Sciences) package program was used to analyze the data. The Shapiro–Wilk test was used to examine whether continuous data showed normal distribution. Descriptive statistics of the data were expressed as mean ± standard deviation for variables showing normal distribution in continuous data. For variables that do not show it, it is stated as [median (min–max)]. For continuous data that did not comply with normal distribution, the Kruskal–Wallis test and the Bonferroni test were used as post hoc tests to compare more than two independent groups. A One-Way ANOVA test was used to compare more than two independent groups in normally distributed data, and the Bonferroni test was used for post hoc analysis. Spearman correlation analysis was used to analyze the relationship between two continuous variables. The significance level was determined as *p* < 0.05.

## 3. Results

The median age of a total of 110 people included in the study was 22.00 (18.00–37.00) years. No statistically significant difference was detected between the median ages of the groups (*p* = 0.825). BMI median values of PCOS patients both receiving metformin treatment and not receiving metformin treatment were higher than the control group (*p* < 0.001). Insulin (*p* < 0.001) and HOMA-IR (*p* < 0.001) score median values of PCOS patients were found to be significantly higher than the control group. The median E2 value of PCOS patients receiving metformin treatment was significantly lower than both the control and untreated PCOS groups (*p* = 0.011). ALT median values of PCOS patients were found to be higher than the control group (*p* = 0.001). The median value of leukocyte count in PCOS patients not receiving metformin treatment was significantly higher than the control group (*p* = 0.016) (Table 1).

When inflammation markers were compared between groups, it was determined that NLR levels did not differ significantly between groups. CRP levels of PCOS patients receiving metformin treatment were found to be higher than both control and PCOS patients not receiving metformin treatment (*p* < 0.001). There was a significant difference between the groups in terms of PLR mean value (*p* = 0.031). The mean PLR value of PCOS patients, both those receiving metformin treatment and those not receiving treatment, was found to be significantly higher than the control group (Table 2).

A significant positive correlation was detected between PLR and NLR levels in the control group (r: 0.658; *p* < 0.001), PCOS + Metformin group (r: 0.485; *p* < 0.001), and untreated PCOS group (r: 0.451; *p*: 0.012). In PCOS patients not receiving metformin treatment, there was a negative significant correlation between NLR and HDL level (r: −0.384; *p*: 0.036), NLR and insulin (r: 0.422; *p*: 0.020), and HOMA-IR score (r: 0.439; *p*: 0.015) (Table 3).

## 4. Discussion

PCOS is a complex endocrine disorder that leads to hyperandrogenemia, insulin resistance, and abnormal glucose and lipid metabolism. It is also thought that PCOS may be a chronic inflammatory disease. The current study found that the BMI, insulin level, and especially the TSH levels of PCOS patients receiving metformin treatment were higher than the control group. The main underlying pathology in PCOS patients is insulin resistance and abdominal obesity [10]. Therefore, obesity and increased insulin secretion are expected findings in PCOS patients compared to the control group. TSH levels of PCOS patients under metformin treatment were found to be higher than the control group. Although previous literature data reported high TPOab levels in PCOS patients, the relationship between PCOS and thyroid dysfunction has not been explained [11]. Meng et al. reported that the prevalence of thyroid disease is higher in patients with insulin resistance [12]. Although the possible relationship between PCOS and thyroid diseases has been demonstrated, new large-scale studies are needed.

In the current study, the leukocyte count of PCOS patients who did not receive metformin treatment was found to be higher than the control group. In addition, the PLR median values of both PCOS patients who received metformin treatment and those who did not receive metformin treatment were significantly higher than the control group. No statistically significant difference was detected between the study groups in terms of NLR median values. Previous studies have associated increased NLR rates with inflammation and complications in chronic diseases [13,14,15]. A previous meta-analysis study showed that NLR was significantly increased in women with PCOS [16]. Another study reported that the levels of inflammation parameters Cystatin C, hs-CRP, and NLR increased in PCOS patients compared to controls [17]. These two studies reported no significant association between PCOS and PLR. Another study examining inflammation markers with PCOS reported that both NLR and PLR were high in patients with PCOS [18]. The current study found PLR levels to be high in PCOS patients, similar to previous studies, but there is no significant difference between groups in terms of NLR levels.

Considering that polycystic ovary syndrome is associated with low-grade chronic inflammation, increased CRP levels can be expected in PCOS patients. In the current study, CRP levels were found to be higher in PCOS patients receiving metformin treatment than in both control and PCOS patients not receiving metformin treatment. A previous meta-analysis study reported increased CRP levels in PCOS patients [19]. Taşkömür et al. reported increased CRP levels in both normal-weight and obese PCOS patients [17]. In the current study, the mean BMI of PCOS patients receiving metformin treatment was higher than both control and PCOS patients not receiving metformin treatment. Liu et al. found NLR, hs-CRP, and MPV levels to be higher in PCOS patients than in controls. Liu et al. reported that CRP levels in obese PCOS patients were higher than in both the non-obese PCOS and control groups [20]. Lee et al. reported that a high BMI was directly related to elevated hs-CRP levels [21]. Moin et al. reported that reported no significant difference in CRP and inflammatory parameters between BMI-matched PCOS and healthy controls [22]. In the current study, no significant difference was found between untreated PCOS patients and controls in terms of BMI. There was also no significant difference between these groups in terms of CRP levels. The current findings support that high CRP in PCOS patients is more closely related to obesity. The current study is similar to previous literature data. In particular, it has been shown that the coexistence of PCOS and obesity is associated with elevated CRP.

In the current study, a significant positive correlation was detected between PLR level and HOMA-IR score, insulin level, and FSH level. There was a significant positive correlation between NLR level and CRP level in PCOS patients receiving metformin treatment. Previous studies have shown that obesity in the abdominal area, increased intra-abdominal fat tissue, low circulating adiponectin levels in PCOS, and deterioration in subcutaneous fat tissue function cause insulin resistance in PCOS patients [10]. Previous studies have reported the association of obesity and inflammation markers with increased HOMA score, insulin resistance, and impaired metabolic parameters [23]. In the current study, high CRP levels in obese PCOS patients may explain the increased insulin resistance and HOMA-IR score. Previous literature data and current findings have shown that inflammation is more prominent, especially in obese PCOS patients. Insulin resistance is also more common in these patients. Studies with a larger population are needed to explain the effect of metformin treatment on both inflammation and insulin resistance in obese PCOS patients.

The current study has some limitations. First, cross-sectional research conducted in a single center may not reflect the general population. PCOS patients were divided into two groups: those receiving treatment and those not receiving treatment. There was a significant difference between the BMI values of PCOS patients. This difference may have affected the results. We recommend that in future studies, PCOS patients who receive treatment and those who do not receive treatment should be matched and compared in terms of obesity. In the patient group receiving metformin treatment, the duration of metformin treatment was ignored. No comments can be made on the possible effects of treatment duration on the study outcomes. Another limitation of the study is that lifestyle factors that may contribute to inflammatory dysregulation, such as proinflammatory diets and gut dysbiosis, were ignored. We recommend that future studies consider confounding factors associated with the proinflammatory process.

The strengths of our study are that the groups were matched in terms of age. We strictly followed the exclusion criteria to minimize the risk of bias in control group selection. Despite the limitations of the study, we believe that determining the relationships of metformin treatment with NLR, PLR, and CRP results in PCOS patients may be pioneering.

## 5. Conclusions

In the current study, PLR was significantly increased in all PCOS patients compared to controls. CRP levels in PCOS patients receiving metformin treatment were significantly higher than both control and untreated PCOS patients. PLR is positively associated with insulin and HOMA-IR scores in PCOS patients. NLR is negatively related to HDL. Current findings suggest that obesity in PCOS patients increases inflammation independent of PCOS. Prospective studies with larger populations are needed to elucidate the relationship of inflammation with metformin treatment and obesity in PCOS patients.

## Figures and Tables

**Table 1:** Comparison of demographic, biochemical, and hematologic parameters between groups.

Variables	Control (*n* = 30)	PCOS + Metformin (*n* = 50)	PCOS (*n* = 30)	*p*	*p* *
Age (years)	23.50 (18.0–35.0)	22.00 (18.0–37.0)	22.00 (18.0–37.0)	0.825 ^b^	
Height (cm)	161.6 ± 4.2	163.4 ± 5.4	162.9 ± 5.8	0.327 ^a^	
Weight (kg)	55.5 ± 8.9 ^1^	71.5 ± 13.1 ^2^	62.7 ± 10.1 ^3^	***<0.001 ^a^***	1–2: ***<0.001*** 1–3: ***0.043*** 2–3: ***0.003***
BMI (kg/m^2^)	21.23 (14.7–30.1) ^1^	25.45 (19.5–44.6) ^2^	22.51 (14.9–33.3) ^3^	***<0.001 ^b^***	1–2: ***<0.001*** 1–3: ***0.023*** 2–3: ***0.006***
Systolic BP (mm/Hg)	120.0 (110.0–130.0)	120.0 (110.0–140.0)	120.0 (110.0–140.0)	0.051 ^b^	
Diastolic BP (mm/Hg)	80.0 (60.0–80.0)	70.0 (60.0–90.0)	70.0 (60.0–90.0)	0.823 ^b^	
Total cholesterol (mg/dL)	161.4 ± 22.7	171.8 ± 31.9	163.3 ± 32.9	0.257 ^a^	
LDL (mg/dL)	89.2 ± 20.9	99.9 ± 27.1	96.2 ± 30.3	0.218 ^a^	
HDL-C (mg/dL)	61.9 ± 16.0	55.5 ± 12.7	62.6 ± 15.3	0.053 ^a^	
Glucose (mg/dL)	87.5 ± 6.9	92.1 ± 11.4	90.4 ± 5.8	0.089 ^a^	
Insulin (µIU/mL)	6.56 (1.01–10.20) ^1^	12.55 (2.67–67.10) ^2^	8.40 (3.04–25.70) ^3^	***<0.001 ^b^***	1–2: ***<0.001*** 1–3: ***0.003*** 2–3: 0.121
HOMA-IR	1.34 (0.45–2.29) ^1^	2.86 (0.49–16.57) ^2^	1.88 (0.68–6.54) ^3^	***<0.001 ^b^***	1–2: ***<0.001*** 1–3: ***0.002*** 2–3: 0.126
TSH	1.44 (0.14–5.13) ^1^	2.24 (0.59–9.00) ^2^	1.45 (0.29–5.79) ^3^	***0.037 ^b^***	1–2: ***0.016*** 1–3: 0.515 2–3: 0.091
DHEA-SO_4_	221.5 (63.3–1815.0)	257.4 (37.4–1100.0)	277.5 (110.0–584.0)	0.201 ^b^	
FSH (IU/L)	7.17 (2.37–16.11)	5.65 (0.05–18.97)	6.31 (1.76.13.79)	0.738 ^b^	
LH (IU/L)	5.35 (1.17–43.64)	6.29 (0.01–35.09)	7.42 (0.01–43.64)	0.179 ^b^	
LH/FSH ratio	0.93 (0.32–3.58)	1.29 (0.01–4.53)	1.73 (0.32–4.48)	0.106 ^b^	
E2 (IU/L)	75.0 (20.40–340.00) ^1^	46.00 (9.90–222.00) ^2^	71.00 (10.10–346.00) ^3^	***0.011 ^b^***	1–2: ***0.006*** 1–3: ***0.031*** 2–3: 0.603
Urea (mg/dL)	22.50 (15.0–49.0)	20.50 (13.0–35.0)	22.50 (12.0–47.0)	0.134 ^b^	
Creatinine (mg/dL)	0.63 ± 0.07	0.65 ± 0.09	0.66 ± 0.09	0.261 ^a^	
ALT (u/L)	12.0 (7.0–31.0) ^1^	16.0 (3.0–67.0) ^2^	14.0 (7.0–48.0) ^3^	***0.001 ^b^***	1–2: ***<0.001*** 1–3: ***0.007*** 2–3: 0.527
AST (u/L)	17.0 (11.0–25.0)	19.0 (11.0–39.0)	17.0 (13.0–48.0)	0.185 ^b^	
HbA1c	5.3 ± 0.4	5.4 ± 0.5	5.2 ± 0.4	0.087 ^a^	
Hemoglobin	13.65 (12.50–15.40)	13.70 (10.00–15.30)	13.90 (12.10–16.20)	0.465 ^b^	
WBC	6865.0 (4250.0–8630.0)	7260.0 (3800.0–12,200.0)	7855.0 (1770.0–11,240.0)	***0.016 ^b^***	1–2: 0.120 1–3: ***0.004*** 2–3: 0.099
Neutrophil	3994.3 ± 1197.4	4322.0 ± 1577.2	4666.3 ± 1557.0	0.217 ^a^	
Lymphocyte	2004.3 ± 498.8	2177.6 ± 584.7	2256.3 ± 553.5	0.197 ^a^	
Plt (10^3^)	256.2 ± 57.4 ^1^	287.1 ± 58.9 ^2^	248.5 ± 79.8 ^3^	***0.021 ^a^***	1–2: ***0.042*** 1–3: 0.646 2–3: ***0.011***

**^a^** One-way ANOVA test; **^b^** Kruskal–Wallis test; ***p*** *: pairwise comparation (Bonferroni test). **CRP**: C-reactive protein; **BMI**: body mass index; **BP**: blood pressure; **HDL-C**: high-density lipoprotein cholesterol; **LDL-C**: low-density lipoprotein cholesterol; **LH**: luteinizing hormone; **FSH**: follicle-stimulating hormone; **ALT**: Alanine aminotransferase; **AST:** Aspartate aminotransferase; **WBC**: white blood cell; **Plt**: platelet. Significant *p* values are highlighted in bold and italics.

**Table 2:** Comparison of inflammatory markers between groups.

Variables	Control (*n* = 30)	PCOS + Metformin (*n* = 50)	PCOS (*n* = 30)	*p*	*p* *
**CRP**	3.10 (2.50–5.00) ^1^	4.05 (1.00–22.60) ^2^	3.20 (3.00–7.30) ^3^	***<0.001 ^b^***	1–2: ***<0.001*** 1–3: 0.336 2–3: ***0.006***
**NLR**	2.11 ± 0.77	2.07 ± 0.83	2.15 ± 0.84	0.917 ^a^	
**PLR**	115.21 ± 41.52 ^1^	138.63 ± 37.71 ^2^	133.06 ± 35.37 ^3^	***0.031 ^a^***	1–2: ***0.009*** 1–3: ***0.043*** 2–3: 0.529

^a^ One-way ANOVA test; ^b^ Kruskal–Wallis test; *p* *: pairwise comparation (Bonferroni test); **CRP**: C-reactive protein; **NLR**: neutrophil to lymphocyte ratio; **PLR:** platelet to lymphocyte ratio. Significant *p* values are highlighted in bold and italics.

**Table 3:** Spearman correlation analysis between variables by groups.

Variables	CONTROL		PCOS + Metformin		PCOS	
	NLR	PLR	NLR	PLR	NLR	PLR
**PLR**	**r: *0.658*** ***p*: *<0.001***		**r: *0.485*** ***p*: *<0.001***		**r: *0.451*** ***p*: *0.012***	
**CRP**	r: −0.187 *p*: 0.322	r: −0.330 *p*: 0.075	**r: *0.378*** ***p*: *0.007***	r: 0.183 *p*: 0.203	r: −0.063 *p*: 0.742	r: −0.055 *p*: 0.771
**T. cholesterol**	r: −0.063 *p*: 0.740	r: −0.330 *p*: 0.075	r: −0.247 *p*: 0.083	r: −0.076 *p*: 0.600	r: −0.092 *p*: 0.627	r: −0.002 *p*: 0.993
**LDL** **(mg/dL)**	r: −0.052 *p*: 0.786	r: −0.041 *p*: 0.829	r: −0.194 *p*: 0.177	r: −0.030 *p*: 0.836	r: −0.058 *p*: 0.762	r: 0.094 *p*: 0.622
**HDL** **(mg/dL)**	r: −0.184 *p*: 0.330	r: −0.195 *p*: 0.303	r: −0.222 *p*: 0.121	r: −0.084 *p*: 0.563	**r: *−0.384*** ***p*: *0.036***	r: −0.341 *p*: 0.066
**Glucose** **(mg/dL)**	r: 0.182 *p*: 0.337	r: −0.062 *p*: 0.747	r: 0.071 *p*: 0.624	r: 0.279 *p*: 0.050	r: −0.180 *p*: 0.342	r: 0.216 *p*: 0.251
**ALT**	r: −0.197 *p*: 0.296	r: 0.118 *p*: 0.534	r: 0.043 *p*: 0.764	r: 0.011 *p*: 0.942	r: −0.177 *p*: 0.351	r: 0.017 *p*: 0.927
**AST**	r: −0.306 *p*: 0.100	r: 0.025 *p*: 0.896	r: −0.099 *p*: 0.493	r: −0.177 *p*: 0.218	r: −0.278 *p*: 0.138	r: 0.006 *p*: 0.977
**Insulin**	r: 0.314 *p*: 0.091	r: 0.158 *p*: 0.404	r: 0.078 *p*: 0.588	r: 0.067 *p*: 0.643	r: 0.248 *p*: 0.187	**r: *0.422*** ***p*: *0.020***
**HbA1c**	r: −0.021 *p*: 0.914	r: −0.143 *p*: 0.452	r: −0.008 *p*: 0.954	r: 0.119 *p*: 0.410	r: −0.210 *p*: 0.266	r: −0.038 *p*: 0.841
**HOMA-IR**	r: 0.353 *p*: 0.056	r: 0.191 *p*: 0.313	r: 0.096 *p*: 0.508	r: 0.107 *p*: 0.458	r: 0.257 *p*: 0.170	**r: *0.439*** ***p*: *0.015***
**TSH**	r: −0.258 *p*: 0.169	r: −0.347 *p*: 0.060	r: −0.244 *p*: 0.088	r: −0.113 *p*: 0.434	r: −0.105 *p*: 0.581	r: 0.036 *p*: 0.850
**DHEA-SO_4_**	r: 0.321 *p*: 0.084	r: 0.223 *p*: 0.236	r: −0.053 *p*: 0.716	r: −0.022 *p*: 0.882	r: 0.133 *p*: 0.484	r: 0.059 *p*: 0.759
**FSH**	r: 0.129 *p*: 0.495	r: 0.176 *p*: 0.352	r: 0.121 *p*: 0.404	***r*: *0.325*** ***p*: *0.021***	r: 0.133 *p*: 0.485	r: 0.255 *p*: 0.174
**LH**	r: 0.229 *p*: 0.223	r: 0.309 *p*: 0.097	r: 0.031 *p*: 0.830	r: −0.032 *p*: 0.827	r: −0.061 *p*: 0.750	r: −0.046 *p*: 0.809
**LH/FSH ratio**	r: 0.064 *p*: 0.736	r: 0.150 *p*: 0.430	r: −0.008 *p*: 0.956	r: −0.244 *p*: 0.088	r: −0.199 *p*: 0.291	r: −0.199 *p*: 0.291
**E2**	r: −0.181 *p*: 0.338	r: 0.122 *p*: 0.521	r: 0.152 *p*: 0.291	r: 0.036 *p*: 0.805	r: 0.018 *p*: 0.926	r: −0.163 *p*: 0.389
**BMI**	r: −0.137 *p*: 0.470	r: 0.040 *p*: 0.834	r: 0.241 *p*: 0.092	r: 0.070 *p*: 0.628	r: 0.322 *p*: 0.083	r: 0.353 *p*: 0.056

Significant *p* values are highlighted in bold and italics.

## Data Availability

The data presented in this study are available on request from the corresponding author due to privacy reasons.
